# Pure Red Cell Aplasia due to B19 Parvovirus Infection after Autologous Stem Cell Transplantation

**DOI:** 10.1155/2011/251930

**Published:** 2011-06-27

**Authors:** Panagiotis Tsirigotis, Konstantinos Girkas, Christina Economopoulou, Anthoula Bouchla, Nikolaos Papanicolaou, Panagiota Economopoulou, Sotirios Papageorgiou, Vassiliki Pappa, John Dervenoulas

**Affiliations:** 2nd Department of Internal Medicine-Propaudeutic, Attikon General University Hospital, University of Athens, P.O. Box 124 62, Rimini-1, Haidari, Athens, Greece

## Abstract

Parvovirus B19 is recognized as a rare cause of pure red cell aplasia (PRCA) in allogeneic stem cell (SCT) and solid organ transplant patients. We report a patient with Hodgkin's disease who developed PRCA due to parvovirus B19 after autologous SCT and who had an excellent response after treatment with gamma-globulin.

## 1. Introduction

Parvovirus B19, the only human pathogenic parvovirus, is highly tropic to human bone marrow and replicates only in erythroid progenitor cells. The basis of erythroid tropism is the tissue distribution of B19 cellular receptor, globoside (blood group P antigen) [1]. Parvovirus is a common infection worldwide. By age 15, approximately 50% of children have detectable virus-specific IgG, evidence of previous infection, while more than 80% of the adults are seropositive [2]. Primary infection is clinically manifested in healthy children as erythema infectiosum or the so-called “fifth disease” while in adults it can produce a syndrome of symmetrical polyarthropathy [3]. In patients with congenital or acquired hemolytic anemia and a short lifespan of red cells, the sudden cessation of erythropoiesis due to B19 infection, usually results in life-threatening anemia characterized as “transient aplastic crisis” [4]. Studies in healthy volunteers experimentally infected with B19 show that after a prodrome of constitutional symptoms, erythroid lineage in bone marrow is transiently depleted followed by a mild anemia that is rarely clinically apparent [3]. Clearance of infection is associated with the appearance of virus-specific neutralizing IgG and IgM antibodies [3]. In immunocompetent patients, the formation of virus-specific antibodies results in lifelong immunity. However, in immunocompromized patients, the inability to produce neutralizing antibodies results in persisting infection and pure red cell aplasia [5].

In this paper, we report a patient with Hodgkin's disease who developed PRCA due to parvovirus B19 infection after autologous SCT.

## 2. Case Presentation

A 48-year-old male, HIV-negative patient with Hodgkin's disease (nodular sclerosis, stage IIB), refractory to 1st line treatment with the ABVD (adriamycin, bleomycin, vinblastine, dacarbazine) regimen, was admitted to our department for further treatment. Salvage treatment with the combination ESHAP (etoposide, solu-medrol, high-dose cytarabine, cisplatin) resulted in complete remission of his disease and was followed by high-dose treatment and autologous transplantation of peripheral blood stem cells.

Conditioning consisted of carmustine, etoposide, cytarabine, and melphalan (BEAM) followed by infusion of PBSC (CD34 = 4.5 × 10^6^/kg). The day of PBSC infusion was considered as day 0. Early posttransplant course was complicated by severe oral and gastrointestinal mucositis and fever without any clinical or laboratory documentation of infection. Engraftment of neutrophils (ANC > 1 × 10^3^/*μ*L) and platelets (PLT > 20 × 10^3^/*μ*L) occurred on day +12 and +15, respectively. Following engraftment, gradual resolution of fever and mucositis was observed, and on day +22 he was discharged in excellent clinical condition. Complete blood counts (CBCs) at discharge on day +22 were as follows: Hb = 11.9 gr/dL, WBC = 5.4 × 10^3^/*μ*L, and PLT = 238 × 10^3^/*μ*L.

On day +30 the patient was admitted in day clinic for evaluation of low-grade fever and generalized pruritus. Clinical examination was unremarkable except for the presence of a generalized maculopapular rash covering the trunk and the upper extremities. Arthralgias and clinical signs of arthritis were both absent. CBC and biochemistry were both normal. Drug reaction was suspected, sulfamethoxazole-trimethoprim (given for pneumocystis jirovecii prophylaxis) was discontinued, and treatment with antihistamines was given and followed by gradual resolution of all clinical symptoms. 

**Figure 1:**
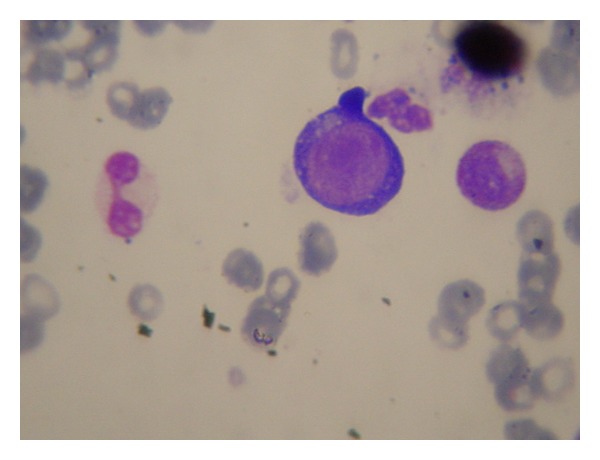
Bone marrow smear: proerythroblast with cytoplasmic vacuolization.

On day +37, he was readmitted to our department because of weakness and severe dyspnea on exertion. Clinical examination was unremarkable except for pallor and tachycardia. CBCs were as follows: Hb = 5.1 gr/dL, WBC = 4.3 × 10^3^/*μ*L with normal differential, and PLT = 150 × 10^3^/*μ*L. Microscopy of the peripheral blood smear showed normal differential and red cell morphology. Biochemistry was normal while reticulocytes were almost absent. Direct Coombs was negative. Bone marrow aspiration showed hypoplastic marrow with almost complete absence of erythroid elements except for the presence of a small number of large proerythroblasts with prominent cytoplasmic vacuolization (Figure 1). Myeloid lineage and megakaryocytes were present with normal maturation and morphology. Testing with quantitative PCR for parvovirus B19 in the peripheral blood revealed the presence of high viral load (1.87 × 10^9^ copies/mL). Remarkably, serological examination for the presence of antibodies IgG and IgM with reactivity against parvovirus B19 were both negative. Based on the above clinical and laboratory findings, the diagnosis of PRCA due to parvovirus infection was established. 

The patient received packed red cell transfusions and high-dose treatment with gamma globulin (IVIg) (0.5 gr/kg daily × 4 days) starting on day +40. A week later, reticulocyte crisis was observed (ret. = 8%) followed by gradual restoration of hemoglobin to normal levels.

Currently, 9 months later, he is in excellent clinical condition, in complete remission of his main disease, and with normal blood counts. Repeat serological testing revealed high titers of IgG-B19 while IgM antibodies were negative.

## 3. Discussion

While the mode of transmission of B19 infection in a normal host is mostly through respiratory tract secretions, it is less well defined in the transplant setting, and primary infection, reinfection, or even reactivation of latent virus have been proposed as possible explanations. Nosocomial transmission in hospitalized patients has been well documented, and patients with aplastic crisis or persistent infection should be considered infectious and need to be isolated. However, patients presented with rash or arthropathy are no longer viremic and should not be considered infectious [6]. Infection can also be transmitted by infected blood products or transplanted organs [6]. Screening of blood samples from blood donors showed that 1 out of 3000 units contained detectable B19 DNA when tested by sensitive PCR techniques [7].

Immunocompromised patients do not have the ability to mount an effective immune response against B19 virus, and persistent infection results in pure red cell aplasia and chronic anemia. IgM and IgG antibodies with specificity against B19 are not detectable, and diagnosis of B19 infection relies on PCR testing for the presence of viral DNA [8]. 

Parvovirus B19 infection is more common among children receiving chemotherapy for acute lymphoblastic leukemia and is associated with prolonged pancytopenia [9]. In solid-organ transplant patients, parvovirus B19 is recognized as a rare cause of PRCA. Review of the literature indicates that approximately 1-2% of all adult solid-organ transplant recipients developed symptomatic active B19 infection during posttransplant course [10]. Although PRCA represents the most common clinical manifestation, the spectrum of B19-associated clinical complications following transplantation is much broader and includes: (1) various cytopenias, or even aplastic anemia, (2) acute hepatic dysfunction ranging from abnormal liver biochemistry to fulminant hepatic failure, (3) myocarditis, (4) thrombotic microangiopathy, (5) chronic allograft dysfunction, or (6) respiratory failure [6, 8].

B19 infection and PRCA is a rare event among allogeneic stem cell transplant (Allo-SCT) recipients. The incidence of B19 infection among allo-SCT recipients as a cause of post-BMT complications was tested in a group of more than 200 patients. During the first 6 months posttransplantation, all patients remained B19-PCR negative. Only 3 cases of B19 infection were diagnosed during the second year of followup. In all cases, severe anemia due to PRCA was the main clinical manifestation of infection [11]. It is reasonable to speculate that the low incidence of documented parvovirus B19 infection may in large part be attributed to the routine use of IVIg in allo-SCT recipients. 

Measurement of anti-B19 IgM and IgG antibodies is at present the standard method for detection of B19 infection in immunocompetent individuals. However, immunosuppressed patients, such as transplant recipients, because of impaired humoral immunity, are not able to mount a detectable antibody response when infected with a virus [6]. In these cases, detection of B19 DNA by PCR analysis is mandatory in order to make a correct diagnosis. B19 DNA is most easily found in bone marrow specimens because of the viral tropism for erythroid precursor cells. However, the specificity may not be high since it has not yet been clarified whether a B19 PCR-positive bone marrow sample is always associated with clinical disease [12]. On the contrary, it seems that detection of B19-DNA in the serum indicates more accurately an ongoing infection, especially when the viral load is high, as it was in the case of our patient [6]. Morphological evaluation of bone marrow smears can aid in the diagnosis of B19 infection. Erythroid hypoplasia, lack of maturation beyond the stage of proerythroblast, and the presence of giant proerythroblasts with intranuclear inclusion or cytoplasmic vacuolization are findings highly suggestive of an ongoing B19 infection [6]. 

No antiviral drug with specificity against B19 parvovirus currently exists. Standard IVIg formulations contain high titers of B19-specific IgG antibodies, and infusion of IVIg has been successfully used in the treatment of B19-infection in immunocompromized patients [13]. Although IVIg treatment is highly effective, relapses are not uncommon, and severely immunosuppressed patients who do not eventually develop their own immunoglobulins remain dependent on maintenance IVIg infusions [5]. 

There is a limited number of papers of PRCA due to parvovirus infection after auto-SCT [14–16]. In conclusion, PRCA due to parvovirus B19 infection is a rare complication following auto-SCT presumably due to the faster immunologic recovery in comparison with allo-SCT or solid-organ transplantation.
